# Using Interpretative Structural Modeling to Identify Critical Success Factors for Safety Management in Subway Construction: A China Study

**DOI:** 10.3390/ijerph15071359

**Published:** 2018-06-28

**Authors:** Ping Liu, Qiming Li, Jing Bian, Liangliang Song, Xiaer Xiahou

**Affiliations:** 1 School of Civil Engineering, Southeast University, Nanjing 211189, China; liupvip@foxmail.com (P.L.); xh@seu.edu.cn (X.X.); 2 School of Civil Engineering, Lanzhou University of Technology, Lanzhou 730050, China; 3 School of Construction Management and Real Estate, Chongqing University, Chongqing 400045, China; 20170301014@cqu.edu.cn; 4 Institute of Engineering Management, Hohai University, Nanjing 211100, China; 230129183@seu.edu.cn

**Keywords:** subway construction, safety, critical success factors, interpretative structural modeling

## Abstract

With the rapid development of urbanization in China, a vast number of subway projects are under construction and planned in many cities. However, the complexities of the environment in subway construction inherently bring about many uncertainties and risk factors. Understanding the inherent properties of the critical success factors (CSFs) will contribute significantly to the subway construction. From this perspective, this paper aims to identify the CSFs for safety management in subway construction. This study screened preliminary CSFs through a literature review and in-depth interviews with experts in China. Based on the data gathered and subsequently analyzed from the questionnaire surveys, a final total of 24 CSFs were identified. Then, interpretative structural modeling was employed to ascertain the interrelations among them. The result showed that the factors concerning the engineering survey and design not only occupy relatively higher scores in the questionnaire survey but also contribute significantly latent impacts on other factors. In addition, the creation of a reasonable schedule and the investment of the safety measures were also found to have a fundamental impact on the safety management of subway construction. This research guided the safety managers in determining the most important safety factors they must face and provided them valuable information that could promote safety performance and decrease the number of accidents over the course of subway construction.

## 1. Introduction

One of the major objectives and tasks for the 13th Five-Year Plan (2016–2020) period in China is to increase the urbanization level from the current 56.1 to 60%. In order to deal with urbanization pressures such as increasing traffic volume and growing demands for land, a vast number of subway projects are under construction and planned in many cities. As a result, as of 31 December 2017, up to 44 cities in China had been approved for the construction of subway systems, and 35 cities had already been operating subway systems. However, the complexity of the environment in subway construction inherently leads to many uncertainties and risk factors, which may lead to accidents [1]. Additionally, subway construction through dense urban areas increases the safety risks on nearby drainage systems, water supply systems, and gas pipelines [2]. Subway construction involves complicated and high-risk construction work. For example, a section of a tunnel collapsed suddenly on 15 November 2008, causing 21 fatalities and 24 injuries [3]. Therefore, it is extremely important to identify the risk factors for safety management in subway construction, which could promote safety performance and decrease the number of accidents.

Safety is the system property or quality, which is necessary and sufficient to ensure that the following construction activities. [4]. According to Hollnagel, most socio-technical systems are intractable, and performance variability is inevitable [5]. Consequently, resilience engineering was employed to enhance the ability of a complex socio-technical system to adapt or absorb disturbance, disruption, and change. [6]. Hollnagel presented the four abilities that characterize resilient systems, which have been widely adopted by resilience engineering researchers: anticipating, monitoring, responding, and learning [5]. A philosophy of safety management, e.g., Safety II, demonstrates the purpose of safety management is the ability to succeed under varying conditions, so that the number of intended and acceptable outcomes is as high as possible [4]. This means that safety is managed by what it achieves (successes, things that go right). In order to do this, safety management cannot only be reactive, it must also be proactive. Currently, safety management in construction projects is divided by different phases. Realizing the influence of design work in subway safety management, Seo and Choi introduced the concept of a safety impact assessment to achieve “design-for-safety” in the design phase [7]. An event tree analysis was applied to quantify the risk at the preliminary design stage in underground construction [8]. Ding et al. developed a safety risk identification system, which aims to assess pre-construction safety risks based on construction drawings [9]. Reducing or eliminating safety risks in the design has been a common trend with great potential in subway projects. Research can also be found in the construction phase. For example, Haslam et al. revealed that front line managers and supervisors are the key individuals in accidents through the investigation of 100 individual construction accidents [10]. 

Since the beginning of modern project management in 1960s, academic researchers have sought to identify a definitive list of Critical Success Factors (CSFs), the key things that project managers must get right in order to deliver a successful product [11]. Previous studies have identified many CSFs for construction project implementation, planning processes, and project risk management [12,13]. Aksorn and Hadikusumo identified 16 CSFs of safety factors and found that management support was the most effective factor [14]. Additionally, Ng et al. tested and validated 31 safety factors to evaluate a contractor’s safety performance at the organization level and the project level [15]. Mahmoudi et al. developed a framework that includes seven main factors and 120 sub factors for the improvement of safety in construction projects and validated the relative importance of the factors [16]. Twenty-five factors affecting construction site safety were identified and summarized into five main factors, consisting of: organizational poor safety awareness of top management, lack of training, poor safety awareness of project managers, reluctance to input resources for safety, and reckless operations [17]. Besides these, Rosa et al. utilized the Functional Resonance Analysis Method (FRAM) to assess risk in sustainable construction and adopted the Analytic Hierarchy Process to reduce the subjectivity in the evaluation of FRAM variability [18]. After an extensive literature review in the field of Resilience Engineering, Patriarca et al. summarized the research in Resilience Engineering and extracted relevant research factors using Factor Analysis and Multi-Dimensional Scaling. [19]. Meanwhile, safety risk factors from contractors and subcontractors [20], safety incentives [21,22], worker’s fatigue-management [23], safety training [17,24,25], safety inspections and job hazard analyses [26,27,28], safety climate [29,30,31], accident and near-miss investigations [32,33,34], and emergency response plans [28,35] were identified as factors influencing the relative effectiveness of CSFs for safety management in the construction phase. 

Most prior studies concentrated on exploring CSFs for safety management in construction, with fewer studies aiming to consider the interrelationships among the CSFs in subway construction. To address this knowledge gap, a method for ascertaining the interrelations among CSFs was proposed. The objective of this paper is to provide a reasonable method to identify the CSFs for safety management in subway construction in order to enhance safety performance. The research objectives include: (1) determining the CSFs for safety management in subway construction; and (2) evaluating the relative importance of CSFs as well as the interrelations among them. The paper is organized and presented in the following structure: Section 2 elaborates on the preliminary screening of factors for safety management in subway construction. A final list of factors is determined through the questionnaire survey method and ISM (Interpretative Structural Modeling) is introduced in Section 3. Section 4 applies this method to identify the most important factors and demonstrates the interrelationships among the factors. Based on the result from Section 4, a further discussion about the findings is conducted in Section 5. Finally, Section 6 draws the overall conclusions.

## 2. Preliminary Screening of Factors for Safety Management in Subway Construction

The research goal of this paper is to explore the CSFs for safety management in subway construction in order to enhance safety management performance. To achieve this purpose, collecting the factors was a critical process of the research, which provided a foundation for the following analysis. This paper puts emphasis on extracting common elements for safety management factors from the activities and processes in subway construction. 

In order to establish a comprehensive list of factors, the factors for safety management in subway construction should be considered from different views. As a construction project always starts with planning and design, followed by a construction stage, decisions made upstream have inherent influences on other stages [36]. Other researchers also found that design work is contributory to lifecycle safety performance in the construction industry [37,38,39]. Therefore, the influencing factors of planning and design should be considered for safety management in subway construction. 

The following two typical options were adopted for the selection of factors. First, a literature review was used to identify the original factors. Second, preliminary factors were selected based on in-depth interviews with experts. Code of construction safety management is a significant part in literatures, which is summary of construction safety management after years’ experience. Therefore, code reading and review is an effective way to acquire factors (see Table 1). Though codes from different counties and regions slightly distinguish in contents and forms, safety management work has something in common. 

According to the literature review, 28 related original factors were found. There were some similarities between the 28 related original factors, so in-depth interviews were then conducted to ensure the reliability of those dimensions and factors. There were some similarities among the 28 related original factors. As a consequence, the experts came to the consensus that the original factors should be reduced to 24 preliminary factors. Finally, a summary of 24 factors are presented in Table 2, which could be grouped into five dimensions based on their properties and attributes: engineering survey and design, construction safety management measures, construction sites security measures, workers’ safety behavior, and guarantee and supervision mechanism. 

## 3. Research Methodology

### 3.1. The In-Depth Interviews

In-depth interviewing is a qualitative research technique, which conducting intensive individual interviews with a small number of respondents to explore their perspectives on a particular idea, program, or situation [44]. In order to ensure the reliability of the preliminary screening of factors, five experienced experts who were engaged in long-term safety management works in subway construction in authoritative enterprises were invited to revise the initial list of factors. All five of experts had over 10 years of working experience and participated in more than three subway construction projects. Considering there were some similarities between the 28 related original factors, the experts came to the consensus that the original factors should be reduced to 24 preliminary factors. Questionnaire survey was refined based on the feedback from the in-depth interviews. 

### 3.2. Questionnaire Survey 

Questionnaire surveys, is widely used method for extracting or identifying key factors [41,45]. The questionnaire was designed to test the preliminary CSFs for safety management in subway construction, in particular from the angle of reasonability and operability. The questionnaire passed the validity and reliability test before being sent out. The survey was conducted in May–August 2017, Six hundred survey questionnaires were distributed to professionals (project employers, contractors, designers, supervisors, and government regulators). They are the main actors in the subway construction process and have a significant influence on safety management. In the first part of the questionnaire, respondents’ personal information was collected, including job title, age, gender, education level, working experience in the subway construction, and other related personal information. In the second part, they were asked to make their own judgments on the CSFs for safety management according to experience in subway construction. A five-point Likert scale was used for data collection about the preliminary CSFs (where 1—can be ignored or not important; 2—slightly important; 3—important; 4—very important; and 5—extremely important).

In total, 600 questionnaires were distributed and 182 of them were collected from project employers, contractors, designers, contractors, and government regulators. Table 3 presents the profile of the data collected via the questionnaires. Among them, over 60% of the respondents have more than 10 years of subway construction safety management experience. Twenty-one questionnaires were discarded and 161 valid questionnaires remained. The 21 questionnaires were found to be invalid due to a significant amount of missing or incomplete data (missing data > 10%) and a very high proportion of same answers. The respondent rate was 26.8%, consistent with the criterion of 20–30% for questionnaire surveys in the construction industry [46,47]. Therefore, this sample was adequate for data analysis. 

### 3.3. Interpretative Structural Modeling (ISM) 

Interpretative structural modeling (ISM) was first proposed by Warfield in 1973, with the aim of analyzing complex systems [48]. Wang et al. applied this method to classify the nine kinds of accident causes into five layers, with definitive relationships between different layers [49]. Tian et al. established the structure of the risk factors at the workplace and reflected the hierarchical relationships of factors [50]. Song et al. made use of ISM to determine the interrelations among vulnerability factors of an urban rail transit system [51]. Considering that subway constructions are continuously affected by many factors, the reason for selecting ISM is that it is a well-established methodology for identifying relationships among specific items, and is frequently used to provide a fundamental understanding of complex situations as well as to put together a course of action for solving a problem [52,53]. 

The ISM process is an interactive process in which a group of both directly related and different elements are organized into an all-inclusive systematic framework [54]. In virtue of the structural relationships diagram, it is easy to visualize the interrelationships between various elements [55]. The steps of ISM development is described below [56,57]:

Step 1: Identify the system factors set. A set of variables affecting the system is defined. The factors for safety management in subway construction were generated using literature review, in-depth interviews, and questionnaire surveys, as shown in Table 2.

Step 2: Construct the adjacency matrix. The adjacency matrix is employed to demonstrate the relationships among the factors in ISM, $a_{ij}$ is the adjacency value of the factor i to j (i=1, 2, …, n; j=1, 2, …, n). A panel of seven experts was invited to participate in making the adjacency matrix, consisting of three professors from Southeast University and four senior managers in Nanjing Metro Co., Ltd. (Nanjing, China). These experts all had more than 10 years of work experience, which could make the judgments more credible and reliable. Before making the judgments, several experts were consulted to ensure that the questions were properly phrased and established. In the process of constructing the adjacency matrix, direct relationships among factors were obtained from the judgments of the experts with the question: does the factor i have an impact on j? As a result, four rounds of discussion were carried out to reach an agreement were about the interrelations. The adjacency matrix is formed by the following three principles:

(1) For the relationships $a_{\mathrm{ij}},$ if i has an impact on j, $a_{\mathrm{ij}}=1$; if not, $a_{\mathrm{ij}}=0$ and vice versa.
(2) If there are strong mutual influences between i and j, then $a_{\mathrm{ij}}$ and $a_{\mathrm{ji}}$ equals 1, if the degree of mutual influence is different between them, then the larger equals 1, the smaller equals 0.
(3) When i=j, then $a_{ij}=a_{ji}=0$.

The adjacency matrix A is as follows:

(1) $$A={\left[\begin{array}{cc} \begin{array}{cc} a_{11} & a_{12}\\ a_{21} & a_{22} \end{array} & \begin{array}{cc} \cdots & a_{1n}\\ \cdots & a_{2n} \end{array}\\ \begin{array}{cc} \vdots & \vdots \\ a_{n1} & a_{n2} \end{array} & \begin{array}{cc} \ddots & \vdots \\ \cdots & a_{nn} \end{array} \end{array}\right]}_{n\times n}$$

Step 3: Generate the reachability matrix. The reachability matrix is used to represent the extent to which different nodes in a directed graph can reach (i.e., indirect influence) each other through certain channels. 

Let

(2) $$E={\left[\begin{array}{cc} \begin{array}{cc} 1 & 0\\ 0 & 1 \end{array} & \begin{array}{cc} \cdots & 0\\ \cdots & 0 \end{array}\\ \begin{array}{cc} \vdots & \vdots \\ 0 & 0 \end{array} & \begin{array}{cc} \ddots & \vdots \\ \cdots & 1 \end{array} \end{array}\right]}_{n\times n}$$

be a n×n identity matrix, the adjacency matrix A and the unit matrix E leads to a new matrix $A_{1}$, and the square of the new matrix $A_{1}$ can be calculated by using the Boolean rules (0+0=0, 0+1=1, 1+1=1, 0×0=1, 0×1=0, 1×1=1), the result is as follows: $A_{2}={\left(A_{1}\right)}^{2}={\left(A+I\right)}^{2}=A^{2}+A+I.$ Through sequential calculations, the reachability matrix R can be calculated by the formula:

(3) $$\left(A+E\right)\neq {\left(A+E\right)}^{2}\neq {\left(A+E\right)}^{3}\neq \cdots \neq {\left(A+E\right)}^{r}={\left(A+E\right)}^{r+1}=R$$

(4) $$R={\left[\begin{array}{cc} \begin{array}{cc} R_{11} & R_{12}\\ R_{21} & R_{22} \end{array} & \begin{array}{cc} \cdots & R_{1n}\\ \cdots & R_{2n} \end{array}\\ \begin{array}{cc} \vdots & \vdots \\ R_{n1} & R_{n2} \end{array} & \begin{array}{cc} \ddots & \vdots \\ \cdots & R_{nn} \end{array} \end{array}\right]}_{n\times n}$$

where $R={\left(A+E\right)}^{r}$ is the reachability matrix of adjacency matrix A.

Step 4: The analysis of the reachability matrix. According to the reachability matrix, the reachability sets and antecedent sets of every factor must be determined. In the i th row $R_{i}$ of reachability matrix R, if $R_{\mathrm{ij}}=1\left(j=1,\;2,\ldots ,\;n\right)$, then the element $R_{\mathrm{ij}}$ is put into the reachable set, which is expressed as $S_{i}$. Meanwhile, in the j th column $R_{j}$ of reachability matrix R, if $R_{\mathrm{ij}}=1\;\left(i=1,\;2,\ldots ,\;n\right)$, then the element $R_{\mathrm{ij}}$ is put into the antecedent sets, which is expressed as $B_{j}$. The intersection of these sets, $S_{i}\cap B_{j}$, is derived for all the factors. If $S_{i}\cap B_{j}=S_{i}$, and then the highest level of factors $L_{1}$ is identified. The column and row corresponding to $L_{1}$ are removed from matrix R. By the same decision rules, $L_{2},{\;L}_{3},\;\ldots ,\;L_{K}$ can be identified. The last step is to establish the hierarchy model of ISM using each level of L.

Step 5: Draw the ISM relationships diagram. In accordance with the results of partitioning the reachability matrix, the top-level factor is positioned at the top of the hierarchy and the second level factor is placed just below the top level. This process is repeated until the bottom level factors are placed at the lowest position in the hierarchy.

### 3.4. Overall Research Methodology

**Figure 1:**
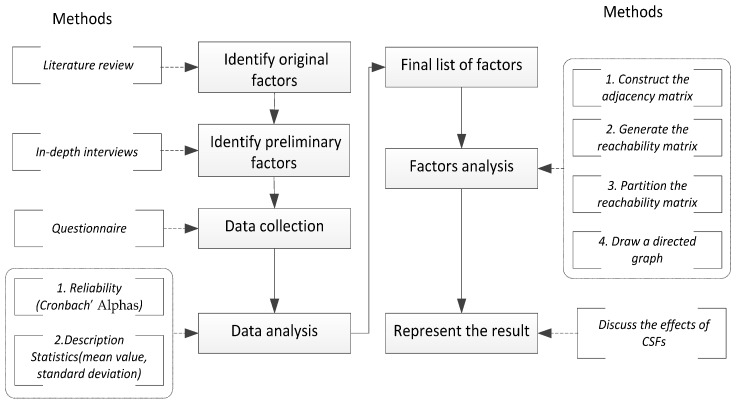
Flowchart of the methodology.

A flowchart of the overall methodology combining the questionnaire survey method and the ISM process adopted here is presented in Figure 1. First, literature review and in-depth interviews methods were used to identify the preliminary factors. Secondly, the final list of factors was then determined through the questionnaire survey method. Then, the ISM was employed to evaluate the relative importance of each factor properly and the interrelations among them. 

## 4. Result Analysis

### 4.1. Result Analysis of Questionnaire Survey

#### 4.1.1. Reliability Analysis 

Reliability analysis was performed on the 161 valid questionnaires, with the results indicating a high reliability (Cronbach’s α = 0.847). Shen et al. noted that the threshold value of Cronbach’s α for a reliable questionnaire is 0.70 [58].

#### 4.1.2. Mean Value and Ranking of the Factors

The scores and rankings of the 24 factors were examined by descriptive statistics (Table 4). If the factor mean value is above 3, it means that it passed the verification. It was found that all 24 factors were critical as they all have mean values above 3. The mean values for these 24 factors range from a minimum of 3.16 (F22) to a maximum of 4.67 (F3). More than half (54%) of the factors’ mean values are over 4.00 (13 factors). This indicates that most of the factors are very important and can be used as CSFs for safety management in subway construction in China. Of the top five factors with the highest mean values, two belong to the dimension of engineering survey and design (D1). The result shows that engineering survey and design for safety management could strongly influence subway construction safety.

**Figure 2:**
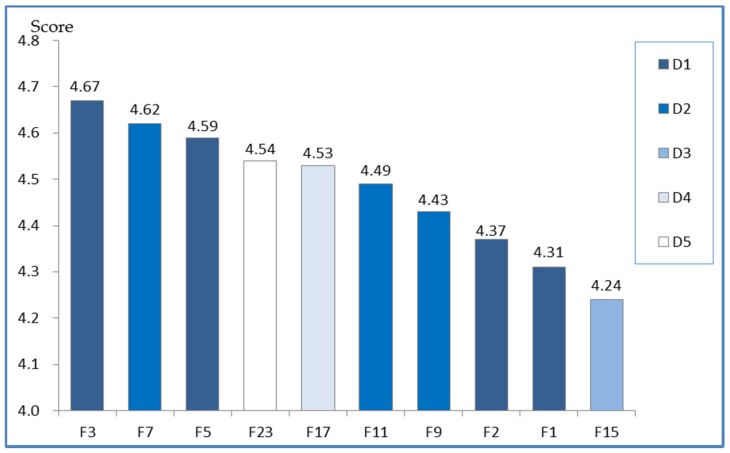
The top ten ranked factors through questionnaire survey analysis.

Based on the questionnaire survey data gathered and the comprehensive analysis of the data, 24 factors were identified as the CSFs for safety management in subway construction. After calculating and analyzing the questionnaire survey data, it was found that the total effects of the dimension layers on safety management in subway construction ranged from 3.57 to 4.27. Given that four out of the 10 top factors were located in the engineering survey and design dimension (Figure 2), it is believed that the role of the engineering survey and design phase in subway construction is a vital factor of successful safety management. Surveying surrounding buildings and municipal pipelines (F3, 4.67), safety training (F7, 4.62), design scheme constructability review (F5, 4.59), ensuring the investment of the safety measures (F23, 4.54), and the foreman’s safety attitude (F17, 4.53) were identified as the most important factors for safety management in subway construction; these were the top five factors, indicating that these factors played vital roles in safety management.

### 4.2. Result Analysis of ISM

**Figure 3:**
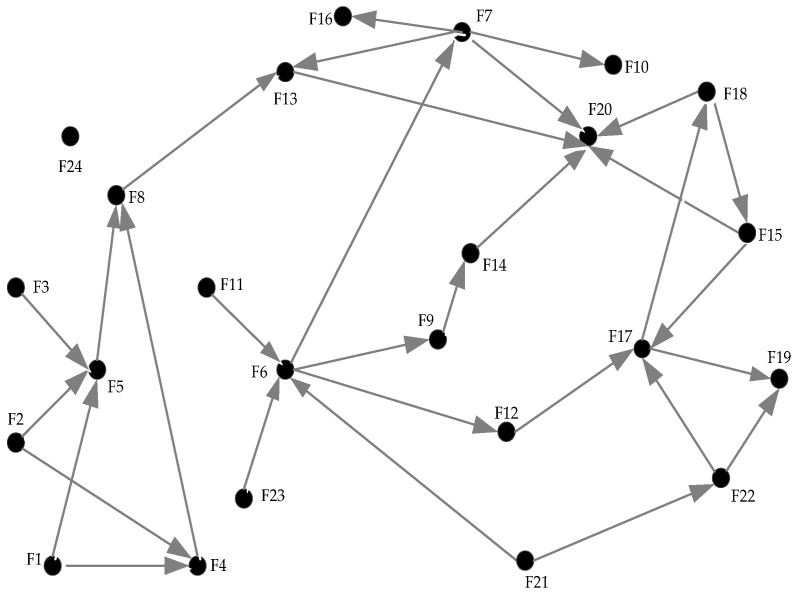
Relationships among factors.

The adjacency matrix and reachability were obtained as per the steps and rules discussed in ISM methodology Section 3.2. Figure 3 graphically presents these relationships, in which nodes represent the safety factors, arrows indicate their interrelations, and the arrow points to the affected node. It is clear that 31 pairs of direct relationships exist among the factors and no bidirectional relationship exists. Only one node (F24) is isolated from the other nodes, and the nodes F6, F7, F17, and F20 exhibit the strongest relationships. However, the indirect interrelations among the safety factors and how these factors influence subway construction remain obscure from this picture.

According to Figure 3, the adjacency matrix A was simultaneously generated based on the relationships among the factors. A reachability matrix M was used to represent the extent to which different nodes in a directed graph can reach each other through certain channels. The feature of transformation means that if there is one channel through which factor Fi can reach Fj directly, there is also one channel through which Fj can reach Fk. Therefore, there must be two channels through which Fi can reach Fk. The calculation process was implemented in Matlab (2018a) and the final result is presented in Table 5. All of the indirect impacts are reflected in the reachability matrix as 1* and the original interrelations are expressed as 1 in Table 5.

According to Step 4 for the level partitioning of matrix M in Section 3.2, the reachability sets and antecedent sets of every factor should be determined. The reachability set is composed of all of the related factors that Fi can reach (on which Fi has an impact), which are shown in the second column of Table 6. The antecedent set is the set composed of all of the factors that can reach Fi, which are shown in the third column. The last column is the intersection set, which contains the common factors in the reachability and antecedent sets. In Table 6, factors F10, F16, F19, F20, and F24 were found at level 1 and subsequently removed before the next partition. The process was repeated for four times until all factors were well arranged.

**Figure 4:**
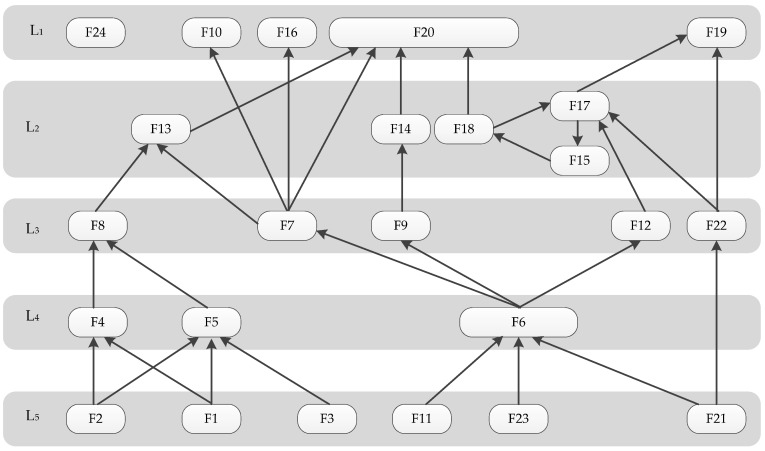
Interpretative structural model.

In accordance with the results of partitioning the reachability matrix, the reachability matrix was rearranged, and the hierarchical structural structure diagram for the safety factors was drawn as a five-layer hierarchy, shown in Figure 4. A relationship between two factors is shown by an arrow which points from a higher-level variable to a lower-level variable. Several observations could be drawn from Figure 4. First, this model is not symmetric. Second, factor F24 is entirely independent and has no relationship with the other factors. Third, factors F1, F2, F3, F11, F21, and F23 are at the deepest layer of the structure. Factors F10, F16, F19, and F20 are at the surface layer of the structure. The rest of the factors are in the middle; moreover, factors F15, F17, and F18 have bidirectional relationships.

## 5. Discussion

Knowing what safety factors influence and how these factors influence subway construction is necessary to promote and improve safety performance level. With the assistance of in-depth interviews and questionnaire survey, a final total of 24 CSFs was identified. Through the analysis of all of the indicator scores, shown in Table 3, it was discovered that the dimension of engineering survey and design (D1) achieved the top score. Consistent with the current studies concerning subway construction safety [59,60], the engineering survey and design dimension (D1) is believed to be the key factor influencing subway construction accidents in China. In addition, four out of the 10 top indicators were located in the engineering survey and design dimension (Figure 2).

Surveying surrounding buildings and municipal pipelines (F3), design scheme constructability review (F5), engineering geological condition analysis, and the disturbance of groundwater (F1) ranked first, third, eighth, and ninth, respectively. Considering that subway constructions through dense urban areas increase the unpredictability of tasks and the relationships of this unpredictability to safety, the above factors represent the crucial tasks in subway construction [2]. The management measures dimension (D2) plays the second most important role in the subway construction safety. Related factors such as safety training (F7) and a reasonable schedule (F11) also obtained higher scores in the final list of factors. Obviously, only with sufficient safety training (F7) can employees work more efficiently and handle unexpected situations more flexibly. While creating a reasonable schedule (F11) is the most important way to guarantee a safe working environment, the stress of meeting the schedule leads to increased safety risks, resulting in increased accidents in subway construction [61]. As the fundamental determinants of human performance, the foreman’s safety attitude (F17) is influenced the safety climate of construction teams [43], which is also a critical factor for decreasing the accidents in subway construction. Moreover, it is worth noting that the factor of ensuring the investment of the safety measures (F23) ranked fourth in the identified 24 factors. It is generally recognized that insufficient safety investment is the immediate cause of accidents [62]. Therefore, safety investment should be strengthened to guarantee safety procedures and a safe operating environment [61]. 

The ISM model (Figure 4) revealed the contextual relationship of identified CSFs and helped develop a hierarchical model. Figure 4 reveals some valuable insights into the relative importance of CSFs as well as the interdependencies among them. In Figure 4, all of the factors associated with subway construction can be classified into five levels. The majority of engineering survey and design factors, such as the disturbance of groundwater (F1), engineering geological condition analysis (F2), and surveying of surrounding buildings and municipal pipelines (F3), are at the deepest hierarchy of the mode, meaning that these factors contribute significantly latent impacts on other dimension factors. The finding is consistent with the studies from Suraji et al. [63] and Haslam et al. [10]. It was found that essential planning and design work are the essential factors, which could bring out inappropriate site conditions and construction operations. Designers can mitigate safety hazards by designing barriers, selecting alternative techniques, and increasing the resilience of the project [64]. In addition, creating a reasonable schedule (F11), establishing a project safety leading group (F21), and ensuring the investment of the safety measures (F23) are also at the bottom of the ISM structure, indicating that they have a fundamental impact on subway construction safety. Consequently, more attention should be paid to these factors. 

Five superficial factors in the upper level have a direct impact on subway construction safety: the supervision of special operations (F10), guaranteeing temporary power use safety (F16), workers’ safety awareness (F19), workers’ potential safety hazard insight (F20), and emergency rescue measures (F24). Once an accident occurs, emergency rescue measures would be undertaken by the experienced project managers according to the accident type and characteristics. Therefore, F24 is isolated from the other factors. These factors will directly affect subway construction safety and cannot influence other factors. Moreover, it is worth noting that workers’ potential safety hazard insight (F20) is influenced by five factors in the middle levels, which means the abilities of the workers to find the potential safety hazards without warning have the most significant impact on reducing accidents in construction [43,65].

The factors in the middle levels ($L_{2}$, $L_{3}$, and $L_{4}$) are influenced by the lower levels and indirectly influence the safety management of subway construction, thus playing a role in connecting the levels above and below. In addition, it could be distinctly observed that safety procedure and policy (F6) has the maximum number of relationships, as it is influenced by factors F11, F21, and F21, and directly influences factors F7, F9, and F12, indicating that this factor plays vital roles in effectively reducing the occurrence of accidents in subway construction. It is recommended that the establishment of appropriate safety procedure and policy is essential in protecting workers from workplace hazards [35].

The Resilience-based Early Warning Indicators (REWI) method was applied to provide early warning to avoid major accidents and to improve the organization’s resilience through the selected set of Contributing Success Factors [66]. The REWI indicators are not static, and provide proactive monitoring and successive evaluating of safety critical activities over time. However, the relationships among the various systematic indicators are not considered. By comparison, the proposed approach in this research was not only to identify CSFs of safety management in subway construction, but also to understand the relationships between different CSFs.

## 6. Conclusions

The promotion of safety management in subway construction is complex, as it involves many uncertainties and a mass of risk factors. Understanding the inherent properties of the CSFs for safety management in subway construction is conducive to retaining a high level of safety performance. This paper provides a comprehensive list of CSFs influencing subway construction safety management in China, based on literature review, in-depth interviews, and questionnaire survey. After calculating and analyzing the questionnaire survey data, it was found that the engineering survey and design phase in subway construction is a vital factor of successful safety management. In addition, surveying surrounding buildings and municipal pipelines, safety training, design scheme constructability review, ensuring the investment of the safety measures, and foreman’s safety attitude were identified as the most important factors for safety management in subway construction; these were the top five factors, indicating that these factors played vital roles in safety management.

Many researchers have tried to identify critical safety factors to effectively prevent construction accidents, while minimal efforts have been made to investigate the relationships and interactions among the CSFs in subway construction. To address this knowledge gap, the interrelations among factors are illustrated by utilizing the proposed ISM method. As the ISM model analysis shows, the engineering survey and design factors, such as surveying surrounding buildings and municipal pipelines, the disturbance of groundwater, and engineering geological condition analysis not only occupy relatively higher scores in the questionnaire survey but are also located at the deepest hierarchy of the interpretative structural model. In addition, the factors of creating a reasonable schedule and ensuring the investment of the safety measures occupy relatively higher scores and are also located at the bottom of the ISM structure. As such, more attention should be paid to these factors. It is also concluded that the factor of workers’ potential safety hazard insight is a critical superficial factor in the upper level, which will directly affect subway construction safety. 

In general, this research contributed to the improvement of subway construction safety management in China. This research guided the participants in determining the most important safety factors to be addressed and provided them with valuable insights into the perception of and knowledge about subway construction safety. Although this study obtained very useful findings regarding safety management in subway construction, more factors should be gathered from construction sites to avoid deviations. Moreover, the research has been raised based on the factors, and their interaction regularity is not quantitatively analyzed, which should be clarified by further research. Meanwhile, subway construction involves many activities and these activities involve many processes, and it is encouraged to apply the proposed approach to analyze the particular activities in future research.

## Figures and Tables

**Table 1:** Codes of construction safety management in different countries or regions.

Country/Region	Code	Abbreviation
China Mainland	Standard for construction safety assessment of metro engineering (GB 50715-2011)	GB 50715
	Code for risk management of underground works in urban rail transit (GB50652-2011)	GB 50652
	Code for Construction company safety manage criterion (GB50656-2011)	GB 50656
	Standard for construction safety inspection (JGJ59-2011)	JGJ59
Hong Kong	Factories and Industrial Undertakings Ordinance (FIUO-Cap.59)	FIUO
	Occupational Safety and Health Ordinance (OSHO-Cap.509)	OSHO
Japan	Construction Occupational Health and Safety Management System (COHSMS)	COHSMS
	Guidelines & COHSMS External System Evaluation	
Singapore	The Factories (Building Operations and Work of Engineering Construction) Regulations	BOWES
	Code of practice for safety management system for construction worksites (Singapore standard CP79:1999)	CP79

**Table 2:** The preliminary list of factors.

Dimensions	Factors	Description	Source
Engineering survey and design (D1)	The disturbance of groundwater (F1)	Analyzes the disturbance of the groundwater table by subway construction	GB 50715, GB 50652
	Engineering geological condition analysis (F2)	Analyzes the main impact of soil physical properties, mechanical properties, and soil distribution during the construction process	GB 50715, GB 50652
	Survey surrounding buildings and municipal pipelines (F3)	Detects the impact of surrounding buildings and complex municipal pipelines on subway construction.	GB 50715, GB 50652
	Construction drawing examination (F4)	Construction drawing in the early stages serves as the foundation for an effective construction program	GB 50652, GB 50656
	Design scheme constructability review (F5)	Checks the rationality and security of the design scheme against the constructability of the subway construction	Moon et al. (2014) [40],
Construction safety management measures (D2)	Safety procedure and policy (F6)	Determines whether there are proper safety procedures and policies for guiding team workers’ safety behavior	FIUO, CP79, BOWES
	Safety training (F7)	A vital factor of a successful safety program is to periodically train and educate all employees to enhance their skills and knowledge about safety at work	JGJ59, FIUO, BOWES, CP79
	Safety technical disclosure (F8)	Disclose all information about the drawings, designs, and safety to the constructors before the construction	GB 50652, JGJ59, FIUO
	Safety checks on construction site (F9)	Safety risk identification and analysis on construction site during subway construction	GB50652, JGJ59, FIUO, BOWES, CP79
	Supervision of special operation (F10)	Special operators must be provided with necessary occupational training and related certificates	GB 50715, JGJ59
	Creating a reasonable schedule (F11)	Avoid the occurrence of accidents caused by rushing to meet the construction schedule	Yu et al. (2014) [41]
	Holding regular safety consultations and meetings (F12)	Participation in regular safety consultations and meetings to discuss work safety	Cheng et al. (2012) [42]
Construction sites security measures (D3)	Personal protective equipment (F13)	Determines whether there is enough proper and available equipment to protect team workers from injury when at work	GB 50656, CP79
	Machinery’s safety state (F14)	Assesses the conditions of construction machines and tools	GB 50715, JGJ59
	Workplace’s safety situation (F15)	Addresses the situation of potential safety hazards, safety accidents, or injuries in the workplace	JGJ59, CP79
	Guaranteeing temporary power use safety (F16)	Determines whether there are proper safety procedures to guarantee temporary power use safety	GB 50715, JGJ59, CP79
Workers’ safety behavior (D4)	Foreman’s safety attitude (F17)	Addresses the foreman’s attitudes toward safety problems and attitudes to promote the workers’ safety skill and knowledge within the construction teams	Li et al. (2017) [43]
	Foreman’s competence (F18)	Addresses the foreman’s experience and competence in dealing with safety problems within his construction team	Li et al. (2017) [43]
	Workers’ safety awareness (F19)	Addresses attitudes to hazards, risks, and the possibility of personal injury in the workplace.	Yu et al. (2014) [41]
	Workers’ potential safety hazard insight (F20)	Assesses capabilities to find or identify the potential safety hazard without warning	Aksorn et al. (2008) [14]
Guarantee and supervision mechanism (D5)	Establishing project safety leading group (F21)	Defines the organizational structure and labor division of safety supervision in subway construction	GB 50715, GB 50656, FIUO
	Safety promotional activities (F22)	Includes safety promotion activities through many mediums such as campaigns, publications, competitions, and contests	Yu et al. (2014) [41]
	Ensuring the investment of the safety measures (F23)	Determines whether the contractor’s safety measures fee is used for subway construction	Yu et al. (2014) [41]
	Emergency rescue measures (F24)	The emergency response plan involves making a plan to follow in the case of a critical situation or severe accident	GB 50656, JGJ59, FIUO, COHSMS, CP79

**Table 3:** Profile of the data collected from the questionnaires.

Respondents’ Characteristics	Description	*N*	%
Project employers (*N* = 46)	Senior manager	27	58.7
	Project manager	19	41.3
Contractors (*N* = 61)	Senior manager	23	37.7
	Project manager	28	45.9
	Safety engineer	10	16.4
Designers (*N* = 17)	Geotechnical engineer	4	23.5
	Designer	13	76.5
Supervisors (*N* = 24)	Chief supervision engineer	13	54.2
	Safety supervision engineer	11	45.8
Government regulators (*N* = 13)	Leader	11	84.6
	Staff	2	15.4
Years of experience (*N* = 161)	<5	23	14.3
	5–9	36	22.4
	10–15	74	45.9
	>15	28	17.4
Regions (*N* = 161)	Nanjing	60	37.2
	Shanghai	45	28.0
	Beijing	22	13.7
	Others	34	21.1

**Table 4:** Scores and ranking of the factors.

Dimension	Factor	Mean	Standard Deviation	Rank	Group Mean	Group Rank	Verification
Engineering survey and design (D1)	F1	4.31	0.62	9	4.27	1	Pass
	F2	4.37	0.87	8			Pass
	F3	4.67	0.96	1			Pass
	F4	3.39	0.76	20			Pass
	F5	4.59	0.81	3			Pass
Construction safety Management Measures (D2)	F6	3.90	0.78	14	4.12	2	Pass
	F7	4.62	0.50	2			Pass
	F8	4.17	1.05	11			Pass
	F9	4.43	0.86	7			Pass
	F10	3.59	1.13	17			Pass
	F11	4.49	0.83	6			Pass
	F12	3.64	0.71	16			Pass
Construction sites security measures (D3)	F13	4.07	1.21	12	3.75	4	Pass
	F14	3.48	0.66	18			Pass
	F15	4.24	0.74	10			Pass
	F16	3.21	0.61	23			Pass
Workers’ safety behavior (D4)	F17	4.53	0.70	5	3.97	3	Pass
	F18	4.01	0.85	13			Pass
	F19	3.88	0.63	15			Pass
	F20	3.47	0.98	19			Pass
Guarantee and Supervision Mechanism (D5)	F21	3.27	1.22	22	3.57	5	Pass
	F22	3.16	0.98	24			Pass
	F23	4.54	0.82	4			Pass
	F24	3.32	1.15	21			Pass

**Table 5:** Reachability matrix.

Factor	F1	F2	F3	F4	F5	F6	F7	F8	F9	F10	F11	F12	F13	F14	F15	F16	F17	F18	F19	F20	F21	F22	F23	F24
F1	1	0	0	1	1	0	0	1*	0	0	0	0	1*	0	0	0	0	0	0	1*	0	0	0	0
F2	0	1	0	1	1	0	0	1*	0	0	0	0	1*	0	0	0	0	0	0	1*	0	0	0	0
F3	0	0	1	0	1	0	0	1*	0	0	0	0	1*	0	0	0	0	0	0	1*	0	0	0	0
F4	0	0	0	1	0	0	0	1	0	0	0	0	1*	0	0	0	0	0	0	1*	0	0	0	0
F5	0	0	0	0	1	0	0	1	0	0	0	0	1*	0	0	0	0	0	0	1*	0	0	0	0
F6	0	0	0	0	0	1	1	0	1	1*	0	1	1*	1*	1*	1*	1*	1*	1*	1*	1*	0	0	0
F7	0	0	0	0	0	0	1	0	0	1	0	0	1	0	1*	1	1*	1*	1*	1*	0	0	0	0
F8	0	0	0	0	0	0	0	1	0	0	0	0	1	0	0	0	0	0	0	1*	0	0	0	0
F9	0	0	0	0	0	0	0	0	1	0	0	0	0	1	1	0	1	1*	1*	1*	0	0	0	0
F10	0	0	0	0	0	0	0	0	0	1	0	0	0	0	0	0	0	0	0	0	0	0	0	0
F11	0	0	0	0	0	1	1*	0	1*	1*	1	1*	1*	1*	1*	1*	1*	1*	1*	1*	0	0	0	0
F12	0	0	0	0	0	0	0	0	0	0	0	1	0	0	1*	0	1	1*	1*	1*	0	0	0	0
F13	0	0	0	0	0	0	0	0	0	0	0	0	1	0	0	0	0	0	0	1	0	0	0	0
F14	0	0	0	0	0	0	0	0	0	0	0	0	0	1	0	0	0	0	0	1	0	0	0	0
F15	0	0	0	0	0	0	0	0	0	0	0	0	0	0	1	0	1	1*	1*	1	0	0	0	0
F16	0	0	0	0	0	0	0	0	0	0	0	0	0	0	0	1	0	0	0	0	0	0	0	0
F17	0	0	0	0	0	0	0	0	0	0	0	0	0	0	1*	0	1	1	1	1*	0	0	0	0
F18	0	0	0	0	0	0	0	0	0	0	0	0	0	0	1	0	1*	1	1*	1	0	0	0	0
F19	0	0	0	0	0	0	0	0	0	0	0	0	0	0	0	0	0	0	1	0	0	0	0	0
F20	0	0	0	0	0	0	0	0	0	0	0	0	0	0	0	0	0	0	0	1	0	0	0	0
F21	0	0	0	0	0	1	1*	0	1*	1*	0	1*	1*	1*	1*	1*	1*	1*	1*	1*	1	1	0	0
F22	0	0	0	0	0	0	0	0	0	0	0	0	0	0	1*	0	1	1*	1*	1*	0	1	0	0
F23	0	0	0	0	0	1	1*	0	1*	1*	0	1*	1*	1*	1*	1*	1*	1*	1*	1*	0	0	1	0
F24	0	0	0	0	0	0	0	0	0	0	0	0	0	0	0	0	0	0	0	0	0	0	0	1

**Table 6:** Level partition of reachability matrix.

Factor	Reachability Set	Antecedent Set	Intersection Set
$L_{1}=\left\{F10,F16,F19,F20,F24\right\}$			
F1	1, 4, 5, 8, 13, 20	1	1
F2	2, 4, 5, 8, 13, 20	2	2
F3	3, 5, 8, 13, 20	3	3
F4	4, 8, 13, 20	1, 2, 4	4
F5	5, 8, 13, 20	1, 2, 3, 5	5
F6	6, 7, 9, 12, 13, 14, 15, 16, 17, 18, 19, 20, 21	6, 11, 21, 23	6, 21
F7	7, 10, 13, 15, 16, 17, 18, 19, 20,	6, 7, 11, 21, 23	7
F8	8, 13, 20	1, 2, 3, 4, 5, 8	8
F9	9, 14, 15, 17, 18, 19, 20	6, 9, 11, 21, 23	9
F10	10	6, 7, 10, 11, 16, 21, 23	10
F11	6, 7, 9, 11, 12, 13, 14, 15, 16, 17, 18, 19, 20	11	11
F12	12, 15, 17, 18, 19, 20	6, 11, 12, 21, 23	12
F13	13, 20	1, 2, 3, 4, 5, 6, 7, 8, 11, 13, 21, 23	13
F14	14, 20	6, 9, 11, 14, 21, 23	14
F15	15, 17, 18, 19 20	6, 7, 9, 11, 12, 15, 17, 18, 21, 22, 23	15, 17, 18
F16	16	6, 7, 11, 16, 21, 23	16
F17	15, 17, 18, 19, 20	6, 7, 9, 11, 12, 15, 17, 18, 21, 22, 23	15, 17, 18
F18	15, 17, 18, 19, 20	6, 7, 9, 11, 12, 15, 17, 18, 21, 22, 23	15, 17, 18
F19	19	6, 7, 9, 11, 12, 15, 17, 18, 19, 21, 22, 23	19
F20	20	1, 2, 3, 4, 5, 6, 7, 8, 9, 11, 12, 13, 14, 15, 17, 18, 20, 21, 22, 23	20
F21	6, 7, 9, 12, 13, 14, 15, 16, 17, 18, 19, 20, 23	21	21
F22	15, 17, 18, 19, 20, 22	21, 22	22
F23	6, 7, 9, 12, 13, 14, 15, 16, 17, 18, 19, 20, 23	23	23
F24	24	24	24
$L_{2}=\left\{F13,F14,F15,F17,F18\right\}$			
F1	1, 4, 8	1	1
F2	2, 4, 5, 8	2	2
F3	3, 5, 8	3	3
F4	4, 8	1, 2, 4	4
F5	5, 8	1, 2, 3, 5	5
F6	6, 7, 9, 12, 13, 14, 15, 17, 18, 21	6, 11, 21, 23	6, 21
F7	7, 13, 15, 17, 18	6, 7, 11, 21, 23	7
F8	8, 13	1, 2, 3, 4, 5, 8	8
F9	9, 14, 15, 17, 18	6, 9, 11, 21, 23	9
F11	6, 7, 9, 11, 12, 13, 14, 15, 17, 18	11	11
F12	12, 15, 17, 18	6, 11, 12, 21, 23	12
F13	13	1, 2, 3, 4, 5, 6, 7, 8, 11, 13, 21, 23	13
F14	14	6, 9, 11, 14, 21, 23	14
F15	15, 17	6, 7, 9, 11, 12, 15, 17, 18, 21, 22, 23	15, 17
F17	17, 18	6, 7, 9, 11, 12, 15, 17, 18, 21, 22, 23	17, 18
F18	15, 18	6, 7, 9, 11, 12, 15, 17, 18, 21, 22, 23	15, 18
F21	6, 7, 9, 12, 13, 14, 15, 17, 18, 23	21	21
F22	15, 17, 18, 22	21, 22	22
F23	6, 7, 9, 12, 13, 14, 15, 17, 18, 23	23	23
$L_{3}=\left\{F7,F8,F9,F12,F22\right\}$			
F1	1, 4, 8	1	1
F2	2, 4, 5, 8	2	2
F3	3, 5, 8	3	3
F4	4, 8	1, 2, 4	4
F5	5, 8	1, 2, 3, 5	5
F6	6, 7, 9, 12, 21	6, 11, 21, 23	6, 21
F7	7	6, 7, 11, 21, 23	7
F8	8	1, 2, 3, 4, 5, 8	8
F9	9	6, 9, 11, 21, 23	9
F11	6, 7, 9, 11, 12	11	11
F12	12	6, 11, 12, 21, 23	12
F21	6, 7, 9, 12, 23	21	21
F22	22	21, 22	22
F23	6, 7, 9, 12, 23	23	23
$L_{4}=\left\{F4,F5,F6\right\}$			
F1	1, 4	1	1
F2	2, 4, 5	2	2
F3	3, 5	3	3
F4	4	1, 2, 4	4
F5	5	1, 2, 3, 5	5
F6	6, 21	6, 11, 21, 23	6, 21
F11	6, 11	11	11
F21	6, 21	21	21
F23	6, 23	23	23
$L_{5}=\left\{F1,F2,F3,F11,F21,F23\right\}$			
F1	1	1	1
F2	2	2	2
F3	3	3	3
F11	11	11	11
F21	21	21	21
F23	23	23	23

## References

[1] Ding L., Zhang L., Wu X., Skibniewski M.J., Yu Q. (2014). Safety management in tunnel construction: Case study of Wuhan metro construction in China. Saf. Sci..

[2] Choi H.H., Cho H.N., Seo J.W. (2004). Risk Assessment Methodology for Underground Construction Projects. J. Constr. Eng. Manag..

[3] Zhou Z., Irizarry J. (2016). Integrated Framework of Modified Accident Energy Release Model and Network Theory to Explore the Full Complexity of the Hangzhou Subway Construction Collapse. J. Manag. Eng..

[4] Hollnagel E. (2014). Safety-I and Safety-II: The Past and Future of Safety Management.

[5] Hollnagel E. (2011). Prologue: The Scope of Resilience Engineering.

[6] Hollnagel E. (2017). Safety-II in Practice: Developing the Resilience Potentials.

[7] Seo J.W., Choi H.H. (2008). Risk-Based Safety Impact Assessment Methodology for Underground Construction Projects in Korea. J. Constr. Eng. Manag..

[8] Hong E.S., Lee I.M., Shin H.S., Nam S.W., Kong J.S. (2009). Quantitative risk evaluation based on event tree analysis technique: Application to the design of shield TBM. Tunn. Undergr. Space Technol..

[9] Ding L.Y., Yu H.L., Li H., Zhou C., Wu X.G., Yu M.H. (2012). Safety risk identification system for metro construction on the basis of construction drawings. Autom. Constr..

[10] Haslam R.A., Hide S.A., Gibb A.G., Gyi D.E., Pavitt T., Atkinson S., Duff A.R. (2005). Contributing factors in construction accidents. Appl. Ergon..

[11] Marais M., Du Plessis E., Saayman M. (2017). A review on critical success factors in tourism. J. Hosp. Tour. Manag..

[12] Zwikael O., Globerson S. (2006). From Critical Success Factors to Critical Success Processes. Int. J. Prod. Res..

[13] Ling F.Y.Y., Sui P.L., Wang S.Q., Lim H.H. (2009). Key project management practices affecting Singaporean firms’ project performance in China. Int. J. Proj. Manag..

[14] Aksorn T., Hadikusumo B.H.W. (2008). Critical success factors influencing safety program performance in Thai construction projects. Saf. Sci..

[15] Ng S.T., Cheng K.P., Skitmore R.M. (2005). A framework for evaluating the safety performance of construction contractors. Build. Environ..

[16] Mahmoudi S., Ghasemi F., Mohammadfam I., Soleimani E. (2014). Framework for Continuous Assessment and Improvement of Occupational Health and Safety Issues in Construction Companies. Saf. Health Work.

[17] Tam C.M., Zeng S.X., Deng Z.M. (2004). Identifying elements of poor construction safety management in China. Saf. Sci..

[18] Rosa L.V., Haddad A.N., Carvalho P.V. (2015). Assessing risk in sustainable construction using the Functional Resonance Analysis Method (FRAM). Cogn. Technol. Work.

[19] Patriarca R., Bergström J., Di G., Costantino F. (2018). Resilience Engineering: Current status of the research and future challenges. Saf. Sci..

[20] Sun Y., Fang D., Wang S., Dai M., Lv X. (2008). Safety Risk Identification and Assessment for Beijing Olympic Venues Construction. J. Manag. Eng..

[21] Wanberg J., Harper C., Hallowell M.R., Rajendran S. (2013). Relationship between Construction Safety and Quality Performance. J. Constr. Eng. Manag..

[22] Hallowell M.R., Hinze J.W., Baud K.C., Wehle A. (2013). Proactive Construction Safety Control: Measuring, Monitoring, and Responding to Safety Leading Indicators. J. Constr. Eng. Manag..

[23] Hinze J., Hallowell M., Baud K. (2013). Construction-Safety Best Practices and Relationships to Safety Performance. J. Constr. Eng. Manag..

[24] Fernández-Muñiz B., Montes-Peón J.M., Vázquez-Ordás C.J. (2007). Safety management system: Development and validation of a multidimensional scale. J. Loss Prev. Process Ind..

[25] Huang X., Hinze J. (2006). Owner’s Role in Construction Safety. J. Constr. Eng. Manag..

[26] Yung P. (2009). Institutional arrangements and construction safety in China: An empirical examination. Constr. Manag. Econ..

[27] Rajendran S., Gambatese J.A. (2009). Development and Initial Validation of Sustainable Construction Safety and Health Rating System. J. Constr. Eng. Manag..

[28] Priyadarshani G.K.K., Jayasuriya S. (2013). Construction Safety Assessment Framework for Developing Countries: A Case Study of Sri Lanka. J. Constr. Dev. Ctries..

[29] Pousette A., Larsson S., Törner M. (2008). Safety climate cross-validation, strength and prediction of safety behaviour. Saf. Sci..

[30] Hon C.K.H., Chan A.P.C., Yam M.C.H. (2014). Relationships between safety climate and safety performance of building repair, maintenance, minor alteration, and addition (RMAA) works. Saf. Sci..

[31] Lyu S., Hon C.K.H. (2018). Relationships among Safety Climate, Safety Behavior, and Safety Outcomes for Ethnic Minority Construction Workers. Int. J. Environ. Res. Public Health.

[32] Wu H.H., Chen H.K., Shieh J.I. (2010). Evaluating Performance Criteria of Employment Service Outreach Program Personnel by DEMATEL Method.

[33] Hinze J., Thurman S., Wehle A. (2013). Leading indicators of construction safety performance. Saf. Sci..

[34] Ghasemi F., Mohammadfam I., Soltanian A.R., Mahmoudi S., Zarei E. (2015). Surprising Incentive: An Instrument for Promoting Safety Performance of Construction Employees. Saf. Health Work.

[35] Ismail Z., Doostdar S., Harun Z. (2012). Factors influencing the implementation of a safety management system for construction sites. Saf. Sci..

[36] Zou Y., Kiviniemi A., Jones S.W. (2016). A review of risk management through BIM and BIM-related technologies. Saf. Sci..

[37] Driscoll T.R., Harrison J.E., Bradley C., Newson R.S. (2008). The Role of Design Issues in Work-Related Fatal Injury in Australia. J. Saf. Res..

[38] Gambatese J.A., Behm M., Rajendran S. (2008). Design’s role in construction accident causality and prevention: Perspectives from an expert panel. Saf. Sci..

[39] Behm M. (2005). Linking construction fatalities to the design for construction safety concept. Saf. Sci..

[40] Moon H., Dawood N., Kang L. (2014). Development of Workspace Conflict Visualization System Using 4D Object of Work Schedule.

[41] Yu Q.Z., Ding L.Y., Zhou C., Luo H.B. (2014). Analysis of factors influencing safety management for metro construction in China. Accid. Anal. Prev..

[42] Cheng E.W.L., Ryan N., Kelly S. (2012). Exploring the perceived influence of safety management practices on project performance in the construction industry. Saf. Sci..

[43] Li Q., Ji C., Yuan J., Han R. (2017). Developing dimensions and key indicators for the safety climate within China ’ s construction teams : A questionnaire survey on construction sites in Nanjing. Saf. Sci..

[44] Boyce C., Neale P., Pathfinder I. (2006). Conducting In-Depth Interviews: A Guide for Designing and Conducting in-Depth Interviews for Evaluation Input.

[45] Li S., Wu X., Zhou Y., Liu X. (2017). A study on the evaluation of implementation level of lean construction in two Chinese firms. Renew. Sustain. Energy Rev..

[46] Zhao X., Singhaputtangkul N. (2016). Effects of Firm Characteristics on Enterprise Risk Management: Case Study of Chinese Construction Firms Operating in Singapore. J. Manag. Eng..

[47] Choudhry R.M., Fang D., Lingard H. (2009). Measuring Safety Climate of a Construction Company. J. Constr. Eng. Manag..

[48] Warfield J.N. (1974). Toward Interpretation of Complex Structural Models. Syst. Man Cybern. IEEE Trans..

[49] Wang C.L., Chao W.U., Shu qing L.I. (2004). Layer Analysis of Accident Causes Based on Interpretation Structure Model. Min. Res. Dev..

[50] Tian Y.Q., Hua L.I., Shang X.G. (2011). Study on Affecting Factors of Risks at Workplace Based on ISM and AHP. China Saf. Sci. J..

[51] Song L., Li Q., List G.F., Deng Y., Lu P. (2017). Using an AHP-ISM Based Method to Study the Vulnerability Factors of Urban Rail Transit System. Sustainability.

[52] Thakkar J., Deshmukh S.G., Gupta A.D., Shankar R. (2007). Development of a balanced scorecard: An integrated approach of Interpretive Structural Modeling (ISM) and Analytic Network Process (ANP). Int. J. Product. Perform. Manag..

[53] Agarwal A., Shankar R., Tiwari M.K. (2007). Modeling agility of supply chain. Ind. Mark. Manag..

[54] Raj T. (2011). Identification and modelling of barriers in the implementation of TQM. Int. J. Product. Qual. Manag..

[55] Venkatesh V.G., Rathi S., Patwa S. (2015). Analysis on supply chain risks in Indian apparel retail chains and proposal of risk prioritization model using Interpretive structural modeling. J. Retail. Consum. Serv..

[56] Han Y., Geng Z., Zhu Q., Lin X. (2015). Energy consumption hierarchical analysis based on interpretative structural model for ethylene production. Chin. J. Chem. Eng..

[57] Han Y., Zhu Q., Geng Z., Xu Y. (2017). Energy and carbon emissions analysis and prediction of complex petrochemical systems based on an improved extreme learning machine integrated interpretative structural model. Appl. Therm. Eng..

[58] Shen Y., Koh T.Y., Rowlinson S., Bridge A.J. (2015). Empirical Investigation of Factors Contributing to the Psychological Safety Climate on Construction Sites. J. Constr. Eng. Manag..

[59] Xiahou X., Yuan J., Li Q., Skibniewski M.J. (2018). Validating DFS concept in lifecycle subway projects in China based on incident case analysis and network analysis. J. Civ. Eng. Manag..

[60] Hansen D.C. (2015). Measuring and Improving Designer Hazard Recognition Skill. Master’s Thesis.

[61] Zhang G., Thai V. (2016). V Expert elicitation and Bayesian Network modeling for shipping accidents: A literature review. Saf. Sci..

[62] Ma Y., Zhao Q., Xi M. (2016). Decision-makings in safety investment: An opportunity cost perspective. Saf. Sci..

[63] Suraji A., Duff A.R., Peckitt S.J. (2001). Development of Causal Model of Construction Accident Causation. J. Constr. Eng. Manag..

[64] Hollnagel E. (2008). Risk + barriers = safety?. Saf. Sci..

[65] Pinto A. (2014). QRAM a Qualitative Occupational Safety Risk Assessment Model for the construction industry that incorporate uncertainties by the use of fuzzy sets. Saf. Sci..

[66] Knut Ø., Kviseth T.R. (2012). Guideline for implementing the REWI method. Resilience Based Early Warning Indicators.

